# The ****β****-SiC Nanowires (~100 nm) Induce Apoptosis via Oxidative Stress in Mouse Osteoblastic Cell Line MC3T3-E1

**DOI:** 10.1155/2014/312901

**Published:** 2014-05-21

**Authors:** Weili Xie, Qi Xie, Meishan Jin, Xiaoxiao Huang, Xiaodong Zhang, Zhengkai Shao, Guangwu Wen

**Affiliations:** ^1^School of Materials Science and Engineering, Harbin Institute of Technology, Harbin 150001, China; ^2^Department of Prosthodontics, School of Stomatology, Harbin Medical University, Harbin 150001, China; ^3^Department of Neurosurgery, The First Affiliated Hospital of Harbin Medical University, Harbin 150001, China; ^4^School of Materials Science and Engineering, Harbin Institute of Technology at Weihai, Weihai 264209, China

## Abstract

Silicon carbide (SiC), a compound of silicon and carbon, with chemical formula SiC, the beta modification (**β**-SiC), with a zinc blende crystal structure (similar to diamond), is formed at temperature below 1700°C. **β**-SiC will be the most suitable ceramic material for the future hard tissue replacement, such as bone and tooth. The *in vitro* cytotoxicity of **β**-SiC nanowires was investigated for the first time. Our results indicated that 100 nm long SiC nanowires could significantly induce the apoptosis in MC3T3-E1 cells, compared with 100 **μ**m long SiC nanowires. And 100 nm long SiC nanowires increased oxidative stress in MC3T3-E1 cells, as determined by the concentrations of MDA (as a marker of lipid peroxidation) and 8-OHdG (indicator of oxidative DNA damage). Moreover, transmission electron microscopy (TEM) was performed to evaluate the morphological changes of MC3T3-E1 cells. After treatment with 100 nm long SiC nanowires, the mitochondria were swelled and disintegrated, and the production of ATP and the total oxygen uptake were also decreased significantly. Therefore, **β**-SiC nanowires may have limitations as medical material.

## 1. Introduction

SiC nanowires are considered to have extensive potential applications due to the excellent mechanical, electrical, and optical properties [1, 2]. During the past decades, efforts have been focused on the synthesis of SiC nanowires. Recently, we fabricated ultra-large-scale SiC nanowires by directly annealing SiOC nanocomposites. Owing to the outstanding physical and chemical stability, SiC nanowires might show good performances in biotechnology and life sciences.

Recent studies have disclosed that nanomaterials are excellent vectors for targeting and delivery of medicines [3, 4]. Nevertheless, the* in vitro* toxicity of SiC nanomaterials has not been investigated. The suitability of nanomaterials must be supported by rigorous studies of their potential toxicity [5, 6]. Thus, in the development of nanomaterials the potential nanomaterial induction of adverse cellular reactions must be considered. Silica nanoparticles, for example, have been demonstrated to have low toxicity if administered in moderate concentration [7]. But, the nanoparticles tend to agglomerate and to lead to protein aggregation at a concentration of 25 *μ*g/mL [8]. Further studies showed that toxic effects of nanowires in HepG2 cells and human epithelial cells only occur at higher concentrations (more than 100 *μ*g/mL) [9]. On the other hand, studies have shown that nanowires readily bind the large protein fibronectin and are readily internalized when incubated with mammalian cells [10].

Osteoblasts synthesize and regulate the deposition and mineralization of the extracellular matrix. Most transformed osteoblast cell lines, although of uniform phenotype, do not exhibit the normal linkage of differentiation and growth arrest [11]. Recent studies have shown that H_2_O_2_, as active oxygen species, had both stimulatory and inhibitory effects on MC3T3-E1 cell viability depending on the concentration and treatment duration [12, 13]. Oxidative stress, resulting from excessive levels of reactive oxygen species, represents a major cause of cellular damage and death in a plethora of pathological conditions including osteoporosis, in which there are markedly increased blood levels of oxidative stress markers [14, 15]. And oxidative stress induced caspase-independent apoptosis of MC3T3-E1 cell* in vitro* [16]. The present study was designed to investigate the SiC nanowires induced apoptosis through ROS generation and oxidative stress via Bax/Bal-2 and caspase pathways in mouse osteoblastic cell line MC3T3-E1. Based on the results described above, we employed TEM to characterize the SiC nanowires before exposure and to investigate the effect of SiC with different lengths on cytotoxicity of MC3T3-E1 cell.

## 2. Materials and Methods

### 2.1. Chemicals

In the present study, *β*-SiC nanowires were synthesized by directly annealing the amorphous SiOC nanocomposites in Ar atmosphere [17]. High-sugar Dulbecco's modified Eagle's medium (DMEM) and fetal bovine serum (FBS) were purchased from Gibco (USA). Penicillin-streptomycin was purchased from Sangon (Shanghai, China). WST-8 was purchased from Dojindo Laboratories (Japan). 3-(4,5-Dimethylthiazol-2-yl)-2,5-diphenyltetrazolium bromide (MTT), 2,7-dichlorodihydrofluorescein diacetate (DCF-DA), propidium iodide (PI), and Hoechst 33342 were purchased from Sigma-Aldrich Chemical Company. Annexin V-FITC Apoptosis Detection Kit I was purchased from BD Biosciences (USA). The primary anti-Bax, anti-Bcl-2, anti-caspase-3, and anti-actin were purchased from Santa Cruz Biotechnology (Santa Cruz, CA, USA).

### 2.2. Preparation and Characterization of SiC Nanowires

The *β*-SiC nanowires were dispersed in deionized (DI) water, phosphate-buffered saline (PBS), or a culture medium and characterized by X-ray diffraction (XRD, Shimadzu XRD-6000) and transmission electron microscopy (TEM, Philips CM12), respectively.

### 2.3. Cell Culture

Preosteoblast subclones (MC3T3-E1) cells were obtained from the Cell Bank of Shanghai Institutes for Biological Sciences, Chinese Academy of Science, China. The cells were cultured in DMEM with 10% FBS, 100 U/mL penicillin, and 100 *μ*g/mL streptomycin at 37°C in humidified environment with 5% of CO_2_.

### 2.4. Cell Morphology Observation

After the incubation, control cells as well as MC3T3-E1 cells treated by different types and concentrations of *β*-SiC nanowires were harvested, gently washed with 0.01 M PBS (pH 7.4), and fixed by 2.5% glutaraldehyde for two hours. Then, the cells were incubated at 37°C for 5 min and embedded into 0.1% agar. The agar was subsequently fixed by 2.5% glutaraldehyde in PBS at 4°C for at least 2 h. The cell samples were washed with PBS and refixed by 1% osmium tetroxide at 4°C for 2 h, dehydrated in epoxy resin [18]. Ultrathin cross sections (~60 nm) of the cells were observed under TEM microscope (Hitachi-7650, Japan).

### 2.5. MTT Assay

Cell viability was determined using a 3-(4,5-dimethylthiazol-2-yl)-2,5-diphenyltetrazolium bromide (MTT) assay. Briefly, the cells were seeded in 96-well dishes at 1 × 10^4^ to 2 × 10^4^ cells per well and pretreated with or without *β*-SiC nanowires for 24 h. Each well was then supplemented with 10 *μ*L of MTT (Sigma) and incubated for 4 h at 37°C. The medium was then removed, and 150 *μ*L of dimethyl sulfoxide (Sigma) was added to solubilize the MTT formazan. The optical density was read at 490 nm.

### 2.6. Apoptosis Assay by Flow Cytometry

Double staining for annexin V-fluorescein isothiocyanate (FITC) and propidium iodide (PI) was carried out with annexin V-FITC apoptosis detection kit. After the treatment, cells were washed twice in cold PBS/sodium azide and centrifuged at 80 ×g for 5 min. The pellets were resuspended in binding buffer at density of 1 × 10^6^ cells/mL. A sample (100 *μ*L) of the solution was transferred to a culture tube and double-stained with 5 *μ*L of annexin V-FITC and 5 *μ*L of PI. After incubation in the dark for 15 min at room temperature, 400 *μ*L of binding buffer was added to the mixture. The intensity of annexin V-FITC and PI was recorded by FACScan flow cytometry (BD Biosciences, USA) and analyzed with Cell Quest software. A total of 10,000 cells in each sample was analyzed and the percentage of positive cells was determined for each histogram.

### 2.7. Nuclear Staining with Hoechst 33342/PI

Hoechst 33342/PI were added to each well and incubated for 10 min at 37°C. The stained cells were visualized under a fluorescent microscope, equipped with a CoolSNAP-Pro color digital camera to examine the degree of nuclear condensation.

### 2.8. Western Blot

Cells were cultured and treated as described above and then lysed with ice-cold lysis buffer containing 50 mmol/L Tris-HCl, pH 7.4; 1% NP-40; 150 mmol/L NaCl; 1 mmol/L EDTA; 1 mmol/L phenylmethylsulphonyl fluoride; and complete proteinase inhibitor mixture (one tablet per 10 mL; Roche Molecular Biochemicals, Indianapolis, IN, USA). After protein content determination using a DC Protein Assay kit (Bio-Rad Laboratories, Hercules, CA, USA) and subsequently incubation with dilute solution (1 : 1000) of primary antibodies including anti-Bax, anti-Bcl-2, anti-caspase3, and anti-actin (Santa Cruz Biotechnology Inc, Santa Cruz, CA). The membranes were then exposed to the secondary antibodies, that is, alkaline phosphatase-labeled goat anti-rabbit immunoglobulin (Santa Cruz), at a dilution of 1 : 1000, followed by exposure to X-ray film [19]. The protein bands were detected using an enhanced chemiluminescence western blotting detection kit (Amersham, Little Chalfont, Buckinghamshire, UK).

### 2.9. Detection of the Level of Superoxide Anion (O_2_^∙−^), MDA, 8-OHdG, and MnSOD

We used the probe dihydroethidium (DHE; Molecular Probes, Eugene, OR, USA) to detect intracellular O_2_^∙−^. O_2_^∙−^ oxidizing DHE to ethidium, which generates a red fluorescent signal. Fluorescence spectrometry of cell O_2_^∙−^ production was performed according to previous methods [20].

Cell samples were prepared as a 10% homogenate in 0.9% saline using a homogenizer on ice according to their respective weight. Then the homogenate was centrifuged, and the supernatant was collected and diluted. The assay of the MDA levels was performed according to the manufacturer's instructions for the MDA detection kit. Competitive ELISA for 8-OHdG was performed according to the manufacturer's protocol. Sample DNA assays were performed in triplicate. Standard 8-OHdG was assayed over a concentration range of 0.125–20 ng/mL in duplicate for each experiment. The average concentration of 8-OHdG per microgram of DNA for each group was calculated for each sample. Controls without added DNA and appropriate blanks were also incorporated into experiments.

The enzyme activity of MnSOD was measured in 5–10 *μ*g of cell protein using a kit. The method utilized tetrazolium salt to quantify O_2_^∙−^ generated by xanthine oxidase and hypoxanthine. The standard curve was generated by using a quality-controlled SOD standard. MnSOD activity was determined by performing the assay in the presence of potassium cyanide to inhibit Cu-ZnSOD, thus measuring the residual MnSOD activity.

### 2.10. ATP Assay

The level of intracellular ATP was determined using the ATP Bioluminescence Assay Kit. Cultured cells were lysed with a lysis buffer, followed by centrifugation at 12,000 ×g for 1 min at 4°C. Finally, the level of ATP was determined by mixing 50 *μ*L of the supernatant with 50 *μ*L of luciferase reagent, which catalyzed the light production from ATP and substrate. The emitted light was linearly related to the ATP concentration and measured by using a microplate luminometer.

### 2.11. Oxygen Consumption

To evaluate the ability of cellular oxygen consumption (VO_2_) during nanowires treatment, the Micro Respirometry System (Strathkelvin, Mitocell S200) was employed to measure oxygen content of culture media. Before the assay, MC3T3-E1 cells were pretreated in low oxygen conditions for 2 h in suspension culture with nanowires.

### 2.12. Statistics

All data was expressed as Mean ± S.D. of three independent experiments. The significance of the variability among the trials was analysed using GraphPad Prism (version 5.0) software.

## 3. Results

### 3.1. Characterization of Nanowires

**Figure 1:**
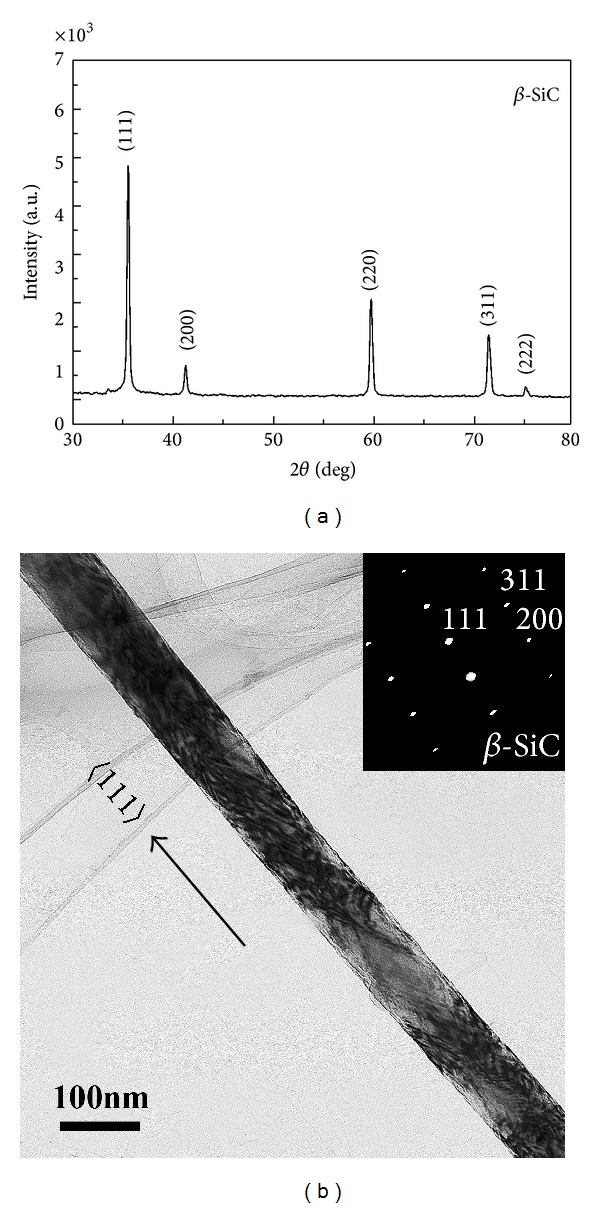
Characterization of nanowires, XRD, and TEM patterns of SiC nanowires. (a) XRD pattern of the SiC nanowires; (b) TEM image of single SiC nanowires and the corresponding SAED pattern.

The size and crystalline structure of nanowires were determined using TEM microscopy and X-ray diffraction (XRD). The average radius of 100 nm long and 100 *μ*m long *β*-SiC nanowires was ~47 nm. The mean length of the two types of nanowires was 100 nm ± 42 nm and 100 *μ*m ± 23 *μ*m, respectively.  Figure 1(a) shows the typical XRD pattern of the SiC nanowires used in our experiments. All the sharp diffraction peaks can be well indexed as *β*-SiC (JCPDS 29-1129). As shown in Figure 1(b), the SiC nanowire was straight and uniform with diameter of 100 nm and the corresponding SAED pattern analysis revealed that the nanowire was *β*-SiC single crystal and the growth direction was along.

### 3.2. 100 nm Long *β*-SiC Nanowires Induced Apoptosis in MC3T3-E1 Cells

**Figure 2:**
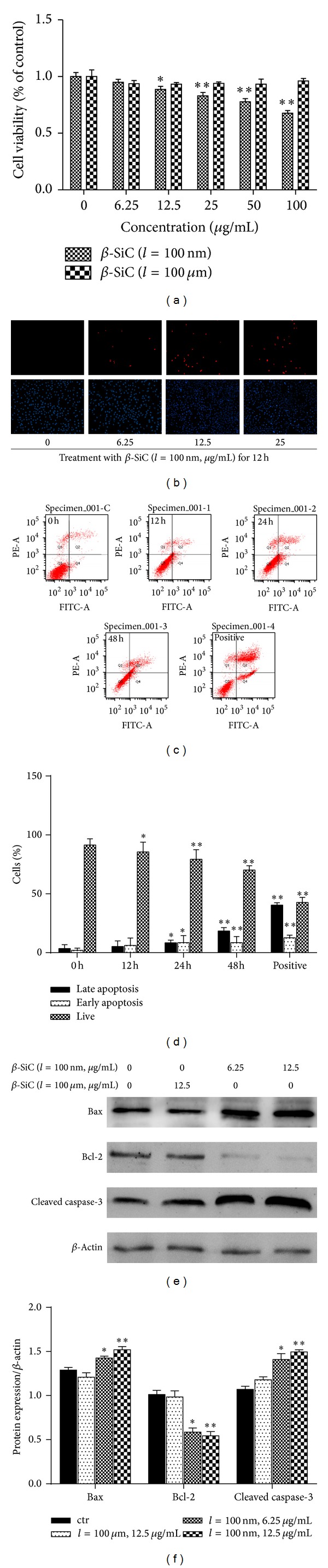
The effect of *β*-SiC nanowires on the cytotoxicity of MC3T3-E1 cells. (a) After 24 h exposure to 6.25, 12.5, 25, 50, and 100 *μ*g/mL of *β*-SiC nanowires (100 nm, 100 *μ*m), MTT assay was used to evaluate the viability of MC3T3-E1 cells. (b) Representative photographs of double staining of PI and Hoechst 33342. The apoptotic cells were observed as PI intense signal after double staining. (c) Cells were stained with annexin V-FITC and PI, analyzed by flow cytometry. H_2_O_2_ treated cells were used as positive control. (d) Cells were treated with 12.5 *μ*g/mL of 100 nm long *β*-SiC nanowires (100 nm) for 12 h, 24 h, and 48 h. The distribution of viable, early apoptotic, later apoptotic, and necrotic cells was analyzed. The data are expressed as percentage of total cells. (e) The expression of apoptotic genes was analyzed by western blot in MC3T3-E1 cells. Values are the means ± SD (*n* = 3) of three individual experiments. (f) Gray value percentage of target protein and *β*-actin. *P < 0.05, **P < 0.01 versus ctr (0 *μ*g/mL).

Dysfunction induced by the decreased population of cells was regarded as an important factor in the pathogenesis of various metabolic diseases. To investigate the effect of *β*-SiC nanowires on MC3T3-E1 cells, cell viability was determined by using MTT assays. After MC3T3-E1 cells were exposed to *β*-SiC nanowires (100 nm or 100 *μ*m) at 6.25, 12.5, 25, 50, and 100 *μ*g/mL for 24 h, cell viability was only decreased in a concentration-dependent manner of 100 nm long *β*-SiC nanowires (Figure 2(a)). To further examine the effects of 100 nm long *β*-SiC nanowires on cell apoptosis, two different methods were used. Quantitative evaluation of apoptosis through annexin V-FITC/PI staining was analyzed by flow cytometry. As shown in Figure 2(c), the rate of apoptotic cells was raised to 28.5% with the treatment of 100 nm long *β*-SiC nanowires (12.5 *μ*g/mL) for 48 h. Determination of DNA content by Hoechst/PI staining indicated that 100 nm long *β*-SiC nanowires increased the amount of dead cells in MC3T3-E1 cells (Figure 2(b)). And treatment with 100 nm long *β*-SiC for 24 h caused a change of the expression level of Bcl-2, Bax, and caspase-3 proteins as markers of cytokine-induced apoptosis (Figure 2(e)). Thus, *β*-SiC nanowires (length = nm) showed significant cytotoxicity in MC3T3-E1 cells, compared with *β*-SiC nanowires (length = *μ*m).

### 3.3. 100 nm Long *β*-SiC Nanowires Increase Oxidative Stress in MC3T3-E1 Cells

**Figure 3:**
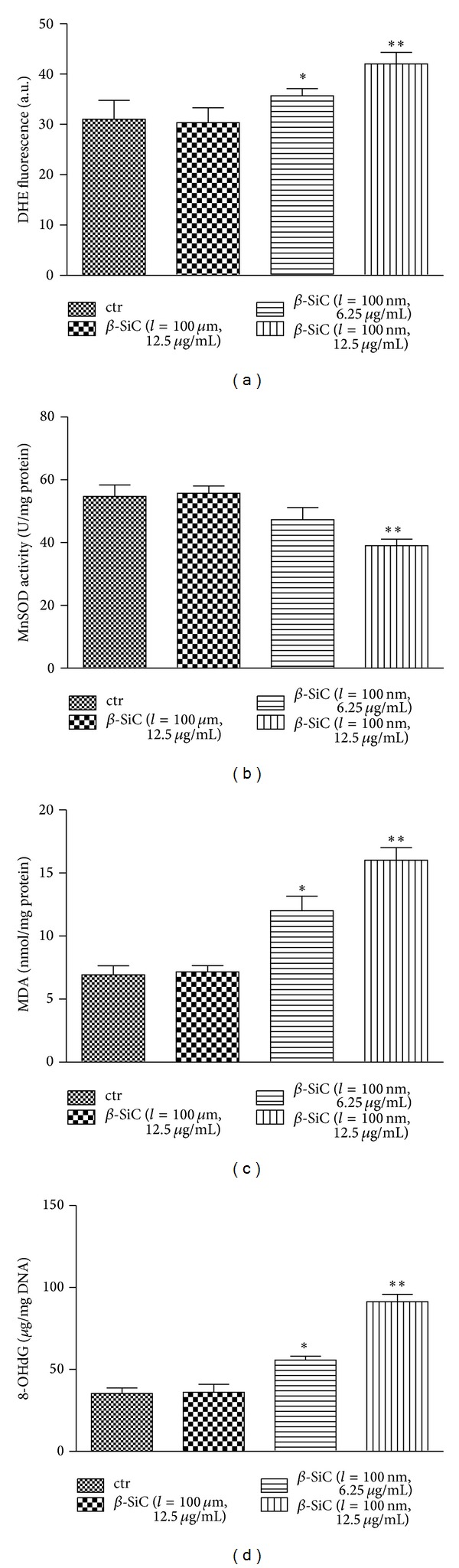
Effects of different *β*-SiC nanowires on oxidative stress in MC3T3-E1 cells. (a) Superoxide level (DHE), (b) MnSOD activity, (c) MDA production, and (d) 8-OHdG level were measured according to the manufacturer's instructions for each assay. Values are the means ± SD (*n* = 3) of three independent experiments. *P < 0.05, **P < 0.01 versus control.

Oxidative stress had been implicated in the pathogenesis of osteocytes apoptosis [21]. To estimate the oxidative effect of different *β*-SiC nanowires, superoxide anion (DHE), MDA, and 8-OHdG levels and MnSOD activity in MC3T3-E1 cells were measured. As an initial indicator of ROS, DHE staining was used, which was a probe for O_2_^∙−^ and produced red fluorescence as a result of the complex between ethidium and DNA as described by previous study [22]. To quantitate changes in O_2_^∙−^ levels, we measured total DHE fluorescence in MC3T3-E1 cell. O_2_^∙−^ levels were induced in the group treated with 100 nm long *β*-SiC nanowires, and 100 *μ*m long *β*-SiC nanowires had no such effects (Figure 3(a)). MnSOD is a pivotal enzyme scavenging ROS* in vivo*. MnSOD activity in MC3T3-E1 cells was significantly increased after treatment with 100 nm long *β*-SiC nanowires (Figure 3(b)). Oxidative stress, as determined by the concentrations of MDA (as a marker of lipid peroxidation) and 8-OHdG (indicator of oxidative DNA damage), remained elevated in the 100 nm long *β*-SiC group. Administration of 100 *μ*m long *β*-SiC could not increase MDA and 8-OHdG levels (Figures 3(c) and 3(d)) in MC3T3-E1 cell. Therefore, 100 nm long *β*-SiC induced oxidative stress in MC3T3-E1 cells.

### 3.4. 100 nm Long *β*-SiC Nanowires Impair Energy Metabolism in MC3T3-E1 Cells

**Figure 4:**
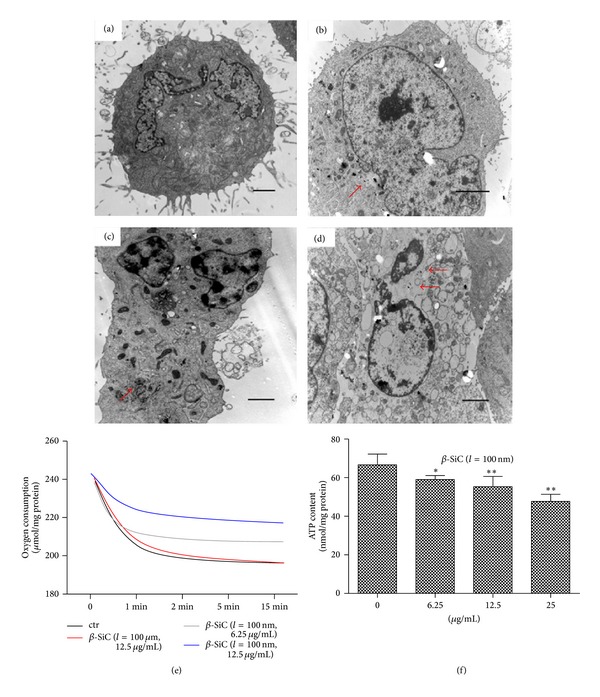
Effects of different *β*-SiC nanowires on energy metabolism in MC3T3-E1 cells. TEM image of *β*-SiC nanowires internalization in MC3T3-E1 cells treated with 12.5 *μ*g/mL of 100 nm long *β*-SiC nanowires ((a)–(d)). Scale bar = 2 *μ*m; red arrows indicate nanowires internalization. Before the oxygen consumption test (e), MC3T3-E1 cells were pretreated in low oxygen conditions for 2 h in suspension culture with different *β*-SiC nanowires. (f) The intracellular ATP concentrations were evaluated after exposure to different *β*-SiC nanowires for 24 h. Values are the means ± SD (*n* = 3) of three independent experiments. *P < 0.05, **P < 0.01 versus control.

Cell apoptosis resulted in decreased energy metabolism and increased production of ROS, especially those linked to mitochondrial signaling events. The TEM images of MC3T3-E1 cells cultured in medium containing *β*-SiC nanowires were shown in Figure 4. All types of cytoplasmic organelles were observed in normal cell (Figure 4(a)) and the cells treated by 100 *μ*m long *β*-SiC nanowires. *β*-SiC nanowires were internalized into plasma, even into the nucleus (Figure 4(b)). The chromatin agglomerated with the microvillus disappeared (Figure 4(c)). Dissolved cytoplasm and swelled endoplasmic reticulum were observed after the cells were treated with *β*-SiC nanowires. The mitochondria were swelled and disintegrated (Figure 4(d)). As 100 nm long *β*-SiC nanowires accelerated the amount of MC3T3-E1 cells, the production of the total oxygen uptake (Figure 4(e)) and ATP (Figure 4(f)) was also reduced significantly, compared with the group treated with 100 *μ*m long *β*-SiC nanowires or the control group. Taken together, it suggested that the mitochondrial signal transduction pathway might be involved in 100 nm long *β*-SiC nanowires induced apoptosis in MC3T3-E1 cells.

## 4. Discussion

Nanoparticles are the particles less than 100 nm in size, which may possess significant health impairments to human bodies [23]. Earlier studies have shown that nanoparticles can penetrate the stratum corneum of epidermis of skin and reach the dermal layer [24]. Nevertheless, the corresponding toxicity of nanoparticles was not tested. The present study was, therefore, designed to measure the cytotoxicity of *β*-SiC nanowires of different sizes* in vitro*.

Compared with* in vivo* studies,* in vitro* assays are less ethically ambiguous and easier to control and reproduce. In the case of* in vitro* assay, it is important to recognize that cell cultures are sensitive to changes in their environment such as fluctuations in pH, temperature, and nutrient and waste concentrations, in addition to the concentration of the potentially toxic agent being tested. As several nanoparticles can adsorb dyes and be redox active, it is also important that the cytotoxicity assay is appropriately selected [25, 26]. Hence, control of the experimental conditions is crucial to ensure that the measured cell changes correspond to the toxicity of the added nanoparticles versus the unstable culture conditions. In our studies, all experiments were performed under controlled conditions. The preliminary MTT assay revealed that *β*-SiC nanowires did not adsorb dyes (data not shown). Apoptosis is a form of programmed cell death which enabled a cell to direct its own destruction. Apoptosis had been evidenced indirectly by three independent methods in our studies (Figure 2). And the expression of related proteins of apoptotic pathway was induced by 100 nm long *β*-SiC nanowires (Figure 2). Oxidative stress was one of the key mechanisms in cellular defense after the uptake of nanoparticles [27–29]. Nanoparticles could induce intracellular oxidative stress by disturbing the balance between the oxidant and antioxidant processes [30, 31]. In the present study, generation and scavenging of free radicals in cells are performed by an oxidation-reduction system containing a plurality of enzymes. Normally, cell mitochondria generate a small quantity of ROS, which is easily removed by antioxidase. However, when free radicals are generated in a large quantity and accumulated through chain reactions, whose situation exceeds the scavenging activity of the antioxidant defense system, the cells are in the state of oxidative stress, thereby causing cytotoxicity damage [32]. MnSOD is a critical link in the free radical scavenging toxification process for cells. We found that, with the increase of concentration of 100 nm long *β*-SiC nanowires, there was a significant decrease in the levels of MnSOD activity, which indicates that the ability to scavenge free radicals of the cells reduces substantially and the cells are in the state of oxidative stress. This study employs MDA as the index for reflecting peroxidation level of lipids in cells. Experimental results show that MDA content of the cells increases substantially with the increase of concentration of 100 nm long *β*-SiC nanowires, which indicates nanowires can induce the cells to generate lipid peroxidation, thereby leading cell function damage and breaking dynamic equilibrium of reactive oxygen and antioxidant substances in the cells. The level of oxidative stress in cells shows substantial correlation with proliferation rate of cells, thus indicating that generation of free radicals and oxidative stress are the main ways for the nanometer materials to cause cytotoxicity damage. As previous study had reported [31], oxidative stress induced transcriptional activation of nuclear respiratory factor 2 (Nrf-2), mitogen activated protein kinase (MAPK), and nuclear factor-*κ*B (NF-*κ*B). Our results indicated oxidative damage to the inner membrane and organelles (Figure 4). We investigated the characterization of *β*-SiC nanowires through TEM and XRD observation. The results of TEM and XRD confirmed the suitability for investigation of actual cytotoxicity of these nanowires. The TEM images also revealed internalization of 100 nm long *β*-SiC nanowires into MC3T3-E1 cells. The *β*-SiC nanowires were enclosed in cellular nucleus and lysosomal, while other nanowires were engulfed. This result is in line with the report that *β*-SiC nanowires could be incorporated into cellular membranes [33]. However, details of the intrusion process and its potential effect on organelles need to be further elucidated. When MC3T3-E1 cells are exposed to *β*-SiC nanowires, we believe, the nanowires are endocytosed from the extracellular fluid: one portion of the plasma membrane is then invaginated and pinched off to form a membrane-bound vesicle.

Increment of permeability in lysosomal membrane is the early event in apoptosis that initiates changes in the mitochondria and activates downstream signal pathway, indicating the active role of lysosomes in apoptosis [34–36]. In present study, however, the increased oxidative stress in cells treated by *β*-SiC nanowires predicted similar apoptotic event that compared with the control group there was a time-dependent increase of necrotic and late apoptotic cells, as reflected by flow cytometry. The similar phenomenon was also revealed by TEM scanning. The damage caused by 100 nm long nanowires was principally mediated through the injury of nucleus and cellular homeostasis, as well as the enhancement of lysosome proliferation via concentration-dependent manner, while the 100 *μ*m long *β*-SiC nanowires induced less cytotoxicity than that of 100 nm long *β*-SiC nanowires. It thus indicates that 100 *μ*m long *β*-SiC nanowires possess favourable biocompatibility and may possess prospect in the biomedical field. Nevertheless, further investigations are required to elucidate the potential cytotoxicity of different cell lines to *β*-SiC nanowires and to assess the biocompatibility and biological safety of such nanowires both* in vitro* and* in vivo*.

## 5. Conclusion

Our results demonstrate that the *β*-SiC nanowires with different lengths result in different cytotoxic effects on MC3T3-E1 cells. As compared with 100 *μ*m long *β*-SiC nanowires, 100 nm long nanowires induced significant increase in the levels of superoxide anion, malondialdehyde, 8-hydroxy-2-deoxyguanosine, and MnSOD (manganese superoxide dismutase) activity. Additionally, 100 nm long nanowires led to apoptosis by oxidative stress in MC3T3-E1 cells.
